# Munchausen syndrome by proxy: a case report

**DOI:** 10.1186/s13256-023-03848-7

**Published:** 2023-04-04

**Authors:** Fadiah Alkhattabi, Israa Bamogaddam, Afaf Alsagheir, Abdullah Al-Ashwal, Raghad Alhuthil

**Affiliations:** Department of Pediatrics, King Faisal Specialist Hospital and Research Centre, Al Takhassousi & 12713, Riyadh, 11211 Saudi Arabia

**Keywords:** Hypoglycemia, Fabricated, Factitious, Munchausen syndrome, Proxy

## Abstract

**Background:**

Inappropriately high levels of insulin secretion can cause the potentially fatal condition of persistent hyperinsulinemic hypoglycemia of infancy. Our paper focuses on another cause of severe hypoglycemia, which can be easily missed.

**Case presentation:**

An 18-month-old Saudi female was referred to our hospital for further investigation and management of her recurrent hypoglycemic attacks as a case of persistent hyperinsulinemic hypoglycemia of infancy. During admission, we noticed multiple red flags from the history; the mother was insisting on a pancreatectomy, rather than going for a positron emission tomography scan, and most importantly, all hypoglycemic attacks occurred while the mother was around. Consequently, after further investigation, the case was diagnosed as a caregiver-fabricated illness, and the case was referred to the Child Protection Center.

**Conclusions:**

One must have a high index of suspicion to diagnose caregiver-fabricated illness. Physicians should be more attentive to prevent such a disease, which could eventually become lethal if left unnoticed.

## Introduction

Persistent hyperinsulinemic hypoglycemia of infancy (PHHI) is a life-threatening condition resulting from inappropriately high levels of insulin secretion. It has a rare incidence of 1:50,000 individuals with a slight male predominance, although, PHHI remains the most common cause of recurrent hypoglycemia in neonates and infants [1]. Early recognition and treatment of this condition are imperative, since severe hypoglycemia can lead to serious neurological deficits and lethal consequences [1, 2]. The two most common causes of this disease are congenital hyperinsulinism (CHI) and insulinoma [3].

Our paper focuses on another cause of severe hypoglycemia, which can be easily missed. We hereby present a case of factitious hypoglycemia in an 18-month-old Saudi female.

## Case presentation

An 18-month-old Saudi female was referred from a local hospital in Najran (a city in southwestern Saudi Arabia) for recurrent hypoglycemic attacks. Prenatal history was unremarkable, except that the mother developed gestational diabetes mellitus. She is a product of full-term cesarean section with a low birth weight of 1 kg and respiratory distress, which required a NICU admission for 2 months. She was developing normally until the age of 9 months when she started to have multiple seizure episodes. The seizures were generalized tonic–clonic with upwards eye rolling and absence of fever, lasting 2–3 min each. The patient has a 6-year-old brother, known to have an epilepsy disorder.

Four months later, the mother started to notice that her daughter is lazy and sleeping for a long time. She experienced her first episode of hypoglycemia in the form of lethargy, drowsiness, and irritability. So, she sought medical advice at their local hospital, where they admitted the patient for observation and discharged her home two days later on dietary recommendations only. As per the mother, her daughter did not improve, in fact, she deteriorated with another episode of seizure and decreased level of consciousness. She was then admitted to the pediatric intensive care unit (PICU) with a blood sugar level of 30 mg/dl and managed with in vitro fertilisation (IVF) only, then she was referred to our hospital (King Faisal Specialist Hospital and Research Centre) in Riyadh for further investigation and management of her recurrent hypoglycemic attacks as a case of PHHI.

Upon arrival at our hospital, the patient was hypoglycemic requiring high dextrose concentrations that reached up to D25%, along with frequent high doses of octreotide and glucagon. The referral notes showed high insulin and low glucose levels with absent C-peptide values. This was confirmed with our blood workup, which showed serum glucose of 4 mmol/L, insulin level of 3744 pmol/L, and low C-peptide values. Multiple samples were taken on different occasions for her frequent hypoglycemia thereafter and showed the same results of low glucose, high insulin, and very low C-peptide levels (Table 1). Physical examination revealed an obvious swelling on the upper and lateral aspect of her right thigh muscle with needle insertion marks, as well as needle marks on her deltoids bilaterally. In addition, the nurse noticed that the child is calm and not crying every time they prick her with the needle.

**Table 1:** Multiple blood workups during admission

Date	Blood glucose (mmol/L)	C-peptide (ng/ml)	Insulin (pmol/L)
29/01	4	0.020	3744
31/01	1.2	0.080	1189
03/02	1.6	0.012	1638
04/02	3.1	0.044	400
06/02	4.3	0.359	615

During admission, we noticed multiple red flags from the history. Furthermore, the mother was insisting on a pancreatectomy rather than going for a positron emission tomography (PET) scan. Finally, and most importantly, all hypoglycemic attacks occurred while the mother was around.

All other investigations were unremarkable, which pushed the diagnosis away from being a normal hyperinsulinemic case and raised the suspicion of an exogenous cause for the hypoglycemia.

While in the PICU, the decision was made by the PICU team, along with the primary team and the child safety team, to keep the patient under close observation with no accompanying family members. During that time, she did not develop any additional hypoglycemic attacks (Table 2). Also, induction of hypoglycemia was demonstrated, and it took her 18 hours to become hypoglycemic with normal critical sample values (Table 3). Therefore, she was discharged from the PICU to the general ward after 5 days observation period.

**Table 2:** Blood glucose level every 2 hours over a 24-hour period

Blood glucose (mmol/L)	4.9	4.6	5.6	4.3	4.7	7	4	5	5.9	4.8	5.1	3.4

**Table 3:** Induced critical sample

Blood glucose (mmol/L)	C-peptide (ng/ml)	Insulin (pmol/L)	ACTH (ng/L)	Cortisol (nmol/L)	GH (ng/ml)
2.2	0.080	8.6	14	136	2.8

The Social Protection Services was informed, and two social workers came to the hospital to investigate the situation. The mother was confronted after that; she made an extensive confession and reported that she had given her daughter insulin injections for more than 6 months (before and during hospital admission) to induce the symptoms. A social inquiry noted that the intention behind her actions was to get the attention of her ex-husband after their recent divorce. Furthermore, both parents live in Najran, the father works while the mother receives monthly support from social affairs. There was no reported history of chronic diseases or psychiatric disorders in either parent.

Therefore, the patient was diagnosed with a caregiver-fabricated illness (CFI), which was, in this case, hypoglycemia, and she was discharged in good health with her maternal grandmother after 19 days of hospital stay under supervision from the Social Protection Services. The father was informed and he agreed to the arrangement.

## Discussion

Caregiver-fabricated illness (CFI), previously known as Munchausen syndrome by proxy (MSBP), is a psychiatric disease in which a person induces intentional harm to another to gain personal benefit from the treatment process [4, 5]. This disease is emerging and becoming more prevalent in children and is now considered a form of child abuse [6]. The goal of the perpetrator, who is frequently the mother, is allegedly to attract the attention of medical professionals and win their sympathy to make up for the psychological neglect, as well as abandonment, they most likely experienced as children [7, 8]. This kind of behavior may also be caused by extrinsic factors. To get her husband’s attention, or avoid having to do things she finds unpleasant, the offending caregiver, for instance, the mother, may play the part of a parent with a sick child when under stress [7, 8]. Similarly, in our case, the mother was the perpetrator; she explained that the reason behind her actions was to get the attention of her ex-husband after their recent divorce. However, the mechanism leading parents to fabricate an illness in their children is still poorly understood.

CFI is extremely challenging to diagnose and frequently confuses medical practitioners because there is no typical presentation, and the symptoms can vary depending on the substance used to fabricate the illness [9]. However, over 400 articles described CFI in the literature, showing the following common presentations: fever, allergic disease, epilepsy, factitious bleeding, renal or gastrointestinal disease, dermatitis artefacta, apnea, and death [3]. Another commonly witnessed presentation of CFI is factitious hypoglycemia. It can be the result of oral antidiabetic drugs or insulin injections. Similarly, in our case, the 18-month-old baby girl was injected with insulin for more than 6 months by her mother. This typical case of CFI was misdiagnosed until reaching our hospital. The diagnosis is most commonly mistaken with persistent hyperinsulinemic hypoglycemia of infancy.

An initial clue to PHHI as a differential diagnosis is an extremely high birth weight averaging around 3.7 kg, with all other physical findings being normal. On the contrary, in our case, the patient was born with a low birth weight of 1 kg. Other diseases, which also present with an increased birth weight include: infants of diabetic mothers and Beckwith-Wiedemann syndrome [10]. Those can be differentiated from PHHI with the presence of other features, such as macroglossia, macrosomia, ear pits, omphalocele, hemihyperplasia, visceromegaly, renal abnormalities, and embryonal tumors in Beckwith-Weidmann syndrome [11]. In PHHI, 75% of infants present in the first 3 months of birth with generalized seizures as the most common feature of their hypoglycemia [12]. Development of PHHI in children beyond 2 years of age is uncommon. PHHI can be divided into two types: focal or diffuse, on the basis of the pancreatic involvement [1].

In regard to the diagnosis, such cases can prove to be very challenging. A high index of suspicion should be present to be able to pick up CFI [13]. Any case of persistent or recurrent illness that cannot be explained medically warrants CFI as a differential diagnosis [4]. History is extremely important. A lot of clues can be picked up through detailed questioning of the caregiver. Usually, the caregiver might mistakenly give away some information when asked about the event at different times [14]. This inconsistency in reporting the details of the incident by the caregiver should not go unnoticed. In fact, it should raise suspicion of possible child abuse [15].

In addition, the occurrence of symptoms only when the caregiver is around the child is another warning sign [4]. Interestingly, our patient did not develop any more hypoglycemic attacks after separating her from her mother, and allowing her grandmother accompany her instead. Cessation of symptoms after separation from the caregiver again hints towards CFI. Also, the physician should keep an eye on any signs of bruises from forceful handling of the child or needle injections during physical examination. In our case, the patient had an obvious swelling on the right thigh with apparent needle markings. Furthermore, the patient was not scared of the needle as any normal child would be. On the contrary, she was very comfortable as if she is used to being injected regularly.

CFI is a diagnosis of exclusion, thus after a complete history and physical examination, all routine workups should be done. This includes complete blood count with differential, renal, liver, and bone profiles. The most important investigations are the measurements of serum glucose, insulin, and C-peptide levels. Typically, endogenous insulin and C-peptide are produced by the beta cells of the pancreas in equal amounts. As a result, high levels of insulin with low C-peptide instantly indicate exogenous insulin administration [16]. Table 1 shows the measurements of our patient. The triad of hypoglycemia, hyperinsulinemia, and low C-peptide supports our diagnosis of the factitious hypoglycemic disorder [17].

Other tests are important for hypoglycemic cases, such as the positron emission tomography (PET) scan, which is used in PHHI to detect any pancreatic abnormalities. Also, genetic testing is now helpful, with more than eight different mutations identified to be associated with congenital hyperinsulinism [18]. The genetic testing and PET scan in our patient were normal. Finally, brain MRI and electroencephalography (EEG) study should be done in such cases to see the effect of the frequent and prolonged hypoglycemia and to rule out brain insult. Both brain MRI and EEG studies were normal in our patient.

Moreover, for PHHI, the treatment is mainly surgical, with either partial or near-total pancreatectomy for focal or diffuse types, respectively [1, 2]. Now, if a patient is misdiagnosed and treated as a case of PHHI, they will be subjected to unnecessary surgery with all its complications and risks and potentially lose a vital organ. This is what happened in the two cases reported by Giurgea *et al.,* 2005, where the diagnosis of CFI was after the partial pancreatectomy in both cases. This could be easily avoided with early identification of CFI [3]. Thankfully, in our case, the patient was diagnosed with CFI before the unnecessary pancreatectomy, and so the management was extremely different. Our main focus was to provide a safe environment for the patient, and therefore, we involved the Child Protection Center in Najran for further evaluation.

Furthermore, the diagnosis of CFI is very challenging. Building rapport and trust between the pediatrician and the patient’s caregiver is an integral part of their job. This trust by itself leads doctors to miss the diagnosis until the child has been seriously harmed [19]. The average time to diagnose CFI usually exceeds 6 months. During that time, the patient is subjected to multiple investigations, which can be inconvenient to the patient and costly for the hospital, thus leading to morbidity and loss of medical resources, respectively [3].

Legal services and child protection services should be involved [6]. In some cases, separation of the child from the caregiver might be necessary. Psychiatric involvement is needed for the perpetrator to prevent further harm. Finally, for comprehensive management, family therapy and marital therapy are indicated [19].

One limitation that should be noted is that since the family is living in Najran, continuous follow-up in our center is difficult; therefore, we referred them to the Child Protection Center in Najran for further follow-up.

## Conclusion

Although multiple cases of induced hypoglycemia have been reported in adults and children, the diagnosis of such cases can still be easily missed. One must have a high index of suspicion in order to diagnose CFI. A good history, with an emphasis on family and social history, along with careful physical examination is the key to reaching the right diagnosis. Physicians should be more attentive to prevent such a disease, which could eventually become lethal if left unnoticed.

## Data Availability

The datasets used in the current study are available from the corresponding author upon reasonable request.
